# Hand hygiene techniques: still a requirement for evidence for practice?

**DOI:** 10.1186/2047-2994-4-S1-O49

**Published:** 2015-06-16

**Authors:** L Price, J Reilly, S Lang, C Robertson, F Cheater, A Chow

**Affiliations:** 1 Department of Nursing & Community Health, Glasgow Caledonian University, Glasgow, UK; 2 Department of Biological & Biomedical Sciences, Glasgow Caledonian University, Glasgow, UK; 3 Department Mathematics & Statistics, Strathclyde University, Glasgow, UK; 4 Faculty of Medicine and Health Sciences, University of East Anglia, Norwich, UK; 5 Department of Clinical Epidemiology, Tan Tock Seng Hospital, Singapore, Singapore

## Introduction

Two hand hygiene techniques are promoted internationally: the World Health Organisation’s 6 step and the Centre for Disease Control’s 3 step techniques; both of which may be considered to have suboptimum levels of empirical evidence for use with alcohol based hand rub (ABHR).

## Objectives

The aim of the study was to to compare the effectiveness of the two techniques in clinical practice.

## Methods

A prospective parallel group randomised controlled trial (RCT) was conducted with 1:1 allocation of 6 step versus the 3 step ABHR hand hygiene technique in a clinical setting. The primary outcome was residual microbiological load. Secondary outcomes were hand surface coverage and duration. The participants were medical and nursing participants (n=120) in a large teaching hospital.

## Results

The 6 step technique was statistically more effective at reducing the bacterial count 1900cfu/ml (95% CI 1300, 2400cfu/ml) to 380cfu/ml (95% CI 150, 860 cfu/ml) than the 3 step 1200cfu/ml (95% CI 940, 1850cfu/ml) to 750cfu/ml (95% CI 380, 1400cfu/ml) (p=0.016) but even with direct observation by two researchers and use of an instruction card demonstrating the technique, compliance with the 6 step technique was only 65%, compared to 100% compliance with 3 step technique. Further those participants with 100% compliance with 6 step technique had a significantly greater log reduction in bacterial load with no additional time or difference in coverage compared to those with 65% compliance with 6 step technique (p=0.01).

## Conclusion

To our knowledge this is the first published RCT to demonstrate the 6 step technique is superior to the 3 step technique in reducing the residual bacterial load after hand hygiene using alcohol based hand rub in clinical practice. What remains unknown is whether the residual bacterial load after the 3 step technique is low enough to reduce risk of transmission from the hands and whether the 6 step technique can be adapted to enhance compliance in order to maximise reduction in residual bacterial load and reduce duration.

## Disclosure of interest

None declared.

